# The administration of clopidogrel attenuates airway hyperresponsiveness and airway inflammation in an ovalbumin specific allergic asthma model

**DOI:** 10.1186/1939-4551-8-S1-A247

**Published:** 2015-04-08

**Authors:** Hoang Kim Tu Trinh, Hae-Sim Park, Yoo Seob Shin, Dong-Hyeon Suh, Jing-Nan Liu

**Affiliations:** 1 Ajou University School of Medicine, South Korea

## Background and objective

Cysteinyl leukotrienes (LTs) contribute to airway inflammation by interacting with type 1 and 2 cysteinyl leukotriene receptors as well as not clearly known type 3 leukotriene receptors. In contrast to LTC4 and LTD4, LTE4 is weak agonist at type 1 and 2 cysteinyl leukotriene receptors. Although LTE4 plays a key role in airway inflammation, our understanding of its potential receptor remains unclear. We investigated the effects of clopidogrel in the development of allergic airway inflammation using mouse asthma model and eosinophil cell-line.

## Methods

BALB/c mice were sensitized by intraperitoneal injection of ovalbumin (OVA) on days 0 and 14, followed by 3 nebulized OVA challenges on days 28-30. On each challenge day, 30mg/kg clopidogrel was administered through intragastric administration 30 minutes before challenge. 48 hours after OVA challenge, mice were assessed for airway hyperresponsiveness (AHR), cell composition and cytokine levels in bronchoalveolar lavage (BAL) fluid. Human eosinophil EOL-1 cells were treated with LTE4 with or without clopidogrel, and intracellular and extracellular eosinophil cationic protein (ECP) expression were investigated by Western blot and ELISA, respectively. Finally, CC ligand 5 (CCL5) levels in BAL fluid were measured by ELISA. Our experiments were approved by Institutional Animal Care and Use Committee of Ajou University (IACUC #152)

## Results

The administration of clopidogrel decreased AHR and airway inflammatory cell numbers including eosinophil in BAL fluid following OVA challenge (*P*<0.01, respectively). These results were associated with decreased levels of Th2 cytokines and CCL5, but not Th1 cytokine in BAL fluid. In histological analysis, the inflammatory cells in peribronchial and perivascular areas as well as mucus-containing goblet cells were also decreased in the clopidogrel administered mice compared to vehicle treated mice (*P*<0.01). LTE4 stimulation decreased intracellular expression of ECP but this expression was attenuated by pre-treatment of clopidogrel.

## Conclusions

Clopidogrel could prevent the development of AHR, airway inflammation, and cytokine production in allergen challenged mice through the inhibition of eosinophilic activation. Clopidogrel could be a novel therapeutic target for asthma treatment.

